# Linking Soil–Orchard Fruit Quality and Circular Food Innovation Through the Valorization of Dried Cherry Pomace in Dairy-Based Spreadable Products

**DOI:** 10.3390/foods15111919

**Published:** 2026-05-29

**Authors:** Mariana Rusu, Irina Gabriela Cara, Iuliana Motrescu, Florina Stoica, Denis Constantin Țopa, Gerard Jităreanu

**Affiliations:** 1 Department of Pedotechnics, Faculty of Agriculture, “Ion Ionescu de la Brad” Iasi University of Life Sciences, 3 Mihail Sadoveanu Alley, 700489 Iasi, Romania; mariana.rusu@iuls.ro (M.R.); florina.stoica@iuls.ro (F.S.); denis.topa@iuls.ro (D.C.Ț.); 2 Research Institute for Agriculture and Environment, “Ion Ionescu de la Brad” Iasi University of Life Sciences, 700490 Iasi, Romania; irina.cara@iuls.ro; 3 Department of Exact Sciences, Faculty of Horticulture, “Ion Ionescu de la Brad” Iasi University of Life Sciences, 700489 Iasi, Romania; iuliana.motrescu@iuls.ro

**Keywords:** soil, cherry orchard, dried cherry pomace, phytochemicals, heavy metals, circular food innovation, by-product valorization

## Abstract

This study explored the link between orchard-derived cherry quality and circular food innovation through the valorization of dried cherry pomace. Sweet cherry fruits from the cultivars Van and Stella, grown under the pedoclimatic conditions of north-eastern Romania, were evaluated for physicochemical traits, phytochemical profile, antioxidant activity, and heavy metal content. In parallel, cherry pomace obtained during juice processing of cultivar Van was freeze-dried, characterized, and incorporated into dairy-based spreadable formulations at 5% and 10% addition levels in order to assess its bioactive potential. The results showed clear cultivar-dependent differences, with Van exhibiting a superior bioactive profile, including higher total polyphenols, flavonoids, anthocyanins, and antioxidant activity than Stella. Heavy metal concentrations in fruits remained below the maximum allowable limits, while health-risk indices indicated no significant non-carcinogenic risk (HI = 3.18 × 10^−2^). The dried cherry pomace powder was characterized by high dietary fiber content (49.83 g/100 g dw), substantial total polyphenols (1046.80 mg GAE/100 g dw), anthocyanins (123.27 mg C3G/100 g dw), and antioxidant activity (21.43 μM TE/g dw). Its incorporation into dairy-based spreadable products significantly improved ash, carbohydrate, fiber, phytochemical content, and antioxidant activity, with the 10% level showing the highest functional enhancement. Sensory evaluation indicated that the 5% formulation achieved the most balanced and preferred overall sensory profile. Overall, the findings support dried cherry pomace as a valuable functional ingredient and highlight a practical circular strategy for reconnecting cherry by-products with value-added food applications.

## 1. Introduction

Sweet cherry (*Prunus avium* L.) is a high-value temperate fruit species of major economic, nutritional, and technological relevance, widely appreciated for its early market availability, sensory attributes, and complex biochemical composition [1]. The fruits are consumed both fresh and processed due to their richness in soluble sugars, organic acids, minerals, vitamin C, anthocyanins, flavonoids, and other phenolic compounds that collectively define their sensory quality and functional properties [2,3]. At the global scale, cherry production is predominantly concentrated in Europe, which accounts for over 65% of total output, estimated at approximately 2.77 million tons [4]. Romania benefits from favorable pedoclimatic conditions for sweet cherry cultivation and remains among the important producing countries, with production particularly concentrated in hilly and sub-Carpathian regions [5].

The performance and quality of sweet cherry production systems are governed by the complex interaction between genotype, orchard management practices, and environmental conditions. Within this framework, soil represents the central regulatory component, as its physical and chemical properties determine water availability, aeration status, nutrient dynamics, and root system functionality, ultimately influencing tree vigor, phenological development, and fruit set [6]. Sweet cherry is particularly sensitive to suboptimal soil conditions such as compaction, poor drainage, water stagnation, and salinity, whereas optimal performance is achieved on well-drained soils with balanced moisture regimes and slightly acidic to neutral pH [7]. In parallel, climatic factors, including solar radiation, temperature, and water availability, modulate physiological processes and fruit ripening, thereby shaping key quality attributes such as fruit size, color, soluble solids content, titratable acidity, pH, mineral composition, and the accumulation of bioactive compounds [6]. Moreover, the transfer and accumulation of essential trace elements such as Zn, Cu, and Fe from soil to fruit further contribute to nutritional value and quality differentiation. Consequently, fruit quality should be understood as an emergent property of the soil–orchard system, reflecting the integrated effects of pedological and climatic drivers throughout the production cycle [8].

In the context of increasing global emphasis on sustainability and circular bioeconomy strategies, the value of fruit production systems extends beyond primary yield and fresh-market performance toward the recovery and functional reintegration of secondary resources. Sweet cherries are directed either to fresh consumption or to industrial processing, depending on their physicochemical characteristics, generating significant amounts of pomace as a by-product. This fraction retains a substantial portion of the fruit’s biochemical profile, including phenolic compounds, pigments, dietary fiber, and minerals of both nutritional and technological relevance [9,10]. The conversion of this material into dried cherry pomace powder represents an efficient strategy for stabilizing bioactive compounds and enabling their incorporation into food matrices, thereby contributing to waste reduction, resource efficiency, and value chain extension within sustainable horticultural systems.

Despite extensive research on sweet cherry production, fruit quality dynamics, and compositional attributes, these domains have largely been investigated independently. As a result, the linkage between soil–orchard system variability, fruit quality expression, and the functional potential of derived by-products within a circular food innovation framework remains insufficiently explored. This gap limits a comprehensive understanding of value continuity across the production chain, from primary production to secondary product valorization.

In addition to their phytochemical richness, cherries and their derived by-products represent a valuable source of dietary fiber, which plays a significant role in promoting gastrointestinal health and supporting a balanced gut microbiome through prebiotic effects. In this context, the incorporation of cherry pomace powder as a bioactive ingredient in dairy-based spreadable products emerges as a promising approach to enhance nutritional value while maintaining desirable sensory and technological properties. However, systematic investigations regarding optimal incorporation levels and functional performance in such matrices remain limited.

Within this framework, the present study aims to establish a conceptual and analytical link between soil–orchard systems and circular food innovation by valorizing dried cherry pomace. Specifically, the study evaluates the relationship between orchard soil conditions, sweet cherry fruit quality, and the valorization of dried cherry pomace as a functional ingredient in dairy-based spreadable products. To this end, sweet cherry fruits were characterized in terms of physicochemical, phytochemical, antioxidant, and heavy metal profiles; dried cherry pomace powder was obtained and characterized; and its incorporation at 5% and 10% levels was assessed in relation to the nutritional, functional, technological, and sensory properties of dairy-based spreadable formulations. The novelty of this work lies in the integration of soil-driven production factors, fruit quality metrics, and by-product valorization into a unified soil–fruit–pomace continuum. This integrative approach contributes to the current state of knowledge by demonstrating how environmental conditions in the orchard influence not only primary fruit quality but also the functional potential of derived by-products, thereby contributing to the development of sustainable and circular agri-food systems.

## 2. Materials and Methods

### 2.1. Study Area and Orchard Conditions

**Figure 1:**
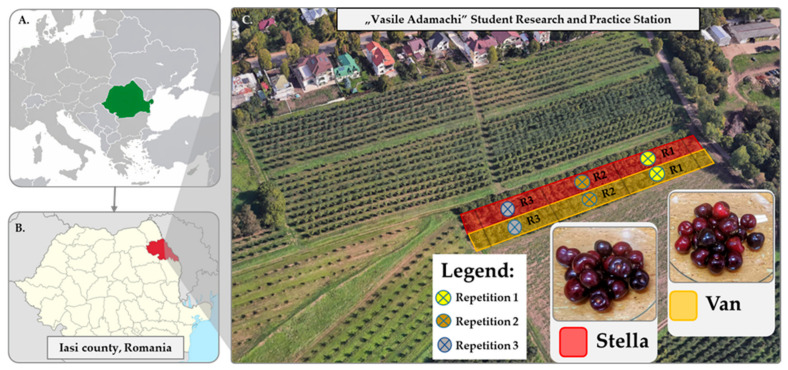
Experimental framework: (**A**) geographical position of Romania within Europe, (**B**) location of Iasi County and the experimental site, and (**C**) aerial view of the experimental orchard with sweet cherry cultivars.

The study was conducted under the pedoclimatic conditions of the North-Eastern region of Romania (Iasi County), at the “Vasile Adamachi” Student Research and Practice Station (47°15′ N, 27°30′ E), part of the Ion Ionescu de la Brad University of Life Sciences, Iasi (IULS) (Figure 1). The experimental site is located in the Jijia Plain (Moldavian Plateau), at elevations of 80–95 m.

During the study period (April–June 2024), the mean air temperature was 18.02 °C, and cumulative precipitation reached 199.6 mm (https://www.fieldclimate.com, accessed on 14 March 2025) [11]. The soil is classified as aric-cambic chernozem (WRB, 2014), with a loamy-clay texture, reflecting the prevailing pedoclimatic conditions that underpin optimal tree growth and fruit development [12].

### 2.2. Biological Material and Experimental Design

Two sweet cherry (*Prunus avium* L.) cultivars, “Van” and “Stella”, were evaluated under orchard conditions representative of the region to assess genotype-driven variability in phenological response. The spatial configuration of the experimental orchard and the evaluated cultivars is presented in Figure 1.

The experiment was organized using a randomized block design with three replicates, each comprising 20 trees/cultivar.

### 2.3. Sample Procedure

Sampling was performed at harvest (June 2024). Fruit samples were collected from randomly selected trees within each replicate, ensuring representative sampling across the canopy.

Fruits were harvested at BBCH 89, rinsed with ultrapure water, air-dried, and stored at −20 °C until analysis.

Soil samples were collected from the 0–20 cm root zone using a soil auger after removal of surface residues. Samples were air-dried, homogenized, and sieved (2 mm) prior to analysis.

### 2.4. Methodology of Determining Physicochemical Parameters of Soil

Soil chemical parameters were determined using standardized analytical procedures widely adopted in soil science and agricultural chemistry.

Soil pH was measured potentiometrically (1:2.5 soil:water) using a calibrated pH meter [13]. Soil organic matter and organic carbon contents were determined by the Walkley–Black dichromate oxidation method [14].

Total nitrogen (N) was determined using the Kjeldahl method [15]. Available potassium (K) was extracted using neutral ammonium acetate and quantified by spectrophotometry. Available phosphorus (P) was extracted with 0.5 M NaHCO_3_ solution at pH 8.5 and determined colorimetrically using the ascorbic acid method [16].

Calcium (Ca) and magnesium (Mg) contents were determined by complexometric titration following extraction with ammonium chloride [17].

### 2.5. Measuring Heavy Metals in Samples

The concentrations of heavy metals (Zn, Cu, Ni, Cd, and Pb) in soil and cherry fruit samples were determined by flame atomic absorption spectrometry (AAS ContrAA 700, Analytik Jena, Jena, Germany) following microwave-assisted acid digestion (SCP Science, Baie-d’Urfé, QC, Canada), in accordance with USEPA Method 3052 [18].

Soil samples (1 g) were digested using a mixture of 6 mL nitric acid (HNO_3_, 65%) and 2.5 mL hydrochloric acid (HCl), whereas fruit samples (1 g) were subjected to digestion with 5 mL nitric acid (HNO_3_, 65%) and 1 mL hydrogen peroxide (H_2_O_2_, 30%), in a Teflon dish at 200 °C for 20 min with a power of 1000 W. After microwave digestion, the resulting solutions were filtered and quantitatively transferred to a final volume of 25 mL using ultrapure water.

Calibration curves were established using multi-element standard stock solutions (1000 mg L^−1^), with working standards prepared in 5% HNO_3_ at five concentration levels (0.05, 0.1, 0.2, 0.25, and 0.5 mg L^−1^). All solutions were prepared using high-purity deionized water. To prevent external contamination, all glassware and laboratory equipment were pre-cleaned with 10% HNO_3_ and thoroughly rinsed with ultrapure water prior to use [19].

### 2.6. Fruit Quality Analysis

Fruit and stone weights and yield/tree were determined using an analytical balance (±0.1 mg) (model BL-600; Sartorius, Göttingen, Germany), and the average values were calculated.

Flesh firmness was assessed using a non-destructive penetrometer (Qualitest HPE), with a 10 cm^2^ surface measuring device, and the results are expressed in N/0, 10 cm^2^ [20].

Total soluble solids (SSC) were measured using a digital refractometer (PR-101, Atago Co., Ltd., Tokyo, Japan) [21].

Titratable acidity (TA) was determined by titration with 0.1 n NaOH (pH 8.1) using phenolphthalein as an indicator, and it was then calculated by determining the amount of NaOH consumed during titration [22].

Fruit pH was measured potentiometrically using a calibrated pH meter (WTW InoLab, Xylem Analytics GmbH, Weilheim, Germany), with prior calibration using standard buffer solutions (pH 4.01 and 7.01) [23]. The vitamin C content of the fruit samples was estimated by quantitative decolorization based on the 2-6-dichlorophenol-indophenoltitration method. Values are expressed in mg/100 g [24].

Color measurements were performed using a Minolta Chroma Meter CR-410 (Konica Minolta, Osaka, Japan), and results were expressed in the CIE L, a, b* color space, where L* represents lightness (0 = black, 100 = white), a* indicates the red–green axis, and b* represents the yellow–blue axis [25]. Derived color parameters were also calculated, including hue angle (h° = arctan (b*/a*)), and chroma (C* = √(a^2^ + b^2^)) [26].

### 2.7. Health-Risk Assessment in Orchard Cherries

#### 2.7.1. Contamination Risk Assessment (ContR)

The contamination risk (ContR) was calculated to quantify soil–fruit metal transfer in relation to food safety using the following equation [27]:

(1) $$\mathrm{ContR}=\frac{C_{f}}{C_{s}}$$

where C_f_ (mg kg^−1^) represents the metal concentration in cherry fruit tissue, and C_s_ (mg kg^−1^) denotes the corresponding concentration in soil.

#### 2.7.2. Health-Risk Assessment of Heavy Metals in Cherry Consumption

The potential health risks associated with the consumption of cherries contaminated with Zn, Cu, Cd, and Pb were evaluated using the following indicators: estimated daily intake (EDI), target hazard quotient (THQ), hazard index (HI), and total carcinogenic risk (TCR) [28]. All abbreviations used in the formulas below are defined in Table 1.

The estimated daily intake (EDI) of metals was calculated using the following Equation (2):

(2) $$EDI=\frac{E_{f}\times E_{d}\times Ir\times Cm}{B_{w}\times TA}$$

The non-carcinogenic risk was assessed using the target hazard quotient (THQ):

(3) $$THQ=\frac{EDI}{R_{f}D}$$

where R_f_D (mg kg^−1^ day^−1^) is the oral reference dose. THQ values < 1 indicate no significant risk, while THQ ≥ 1 suggests a potential risk of adverse health effects, according to USEPA guidelines.

The hazard index (HI), representing the cumulative non-carcinogenic risk, was calculated as:

(4) $$\mathrm{HI}=\Sigma {THQ}_{i}$$

An HI value greater than 1 (HI > 1) indicates a potential health concern due to combined exposure to multiple metals.

The carcinogenic risk (CR) was estimated as:

(5) $$CR=EDI\times C_{sf}\times 10^{-3}$$

where C_sf_ (mg/kg·day)^−1^ is the cancer slope factor. The parameters applied in the equation correspond to the assessment of non-carcinogenic risk, whereas the values used for carcinogenic risk (CR) estimation are presented in Table 1. Risk levels were interpreted according to established thresholds: values below. Risk levels were interpreted according to established thresholds: values below 1 × 10^−6^ are considered to pose no significant health risk; those between 1 × 10^−6^ and 10^−5^ indicate a low risk; values ranging from 1 × 10^−5^ to 10^−4^ are are classified as moderate; risks between 1 × 10^−4^ and 1 × 10^−3^ are regarded as high; and values exceeding 1 × 10^−3^ are considered to represent a very high risk to human health [29].

**Table 1:** Input parameters used for adult carcinogenic risk assessment through cherry consumption.

Parameter	Unit	Adult Value/Assumption	References
Cm (Metal concentration)	mg/kg		[30]
B_w_ (Body weight)	kg	80	
E_f_ (Exposure frequency)	days/year	90	
E_d_ (Exposure duration)	years	70	[31]
Ir (Daily ingestion rate)	kg/person/day	0.154	[32]
TA (Averaging time for carcinogenic effects)	days	25.550 (365 × E_d_)	[33]
R_f_D (Zn)	mg/kg-day	0.37	[34,35]
R_f_D (Cd)	mg/kg-day	0.001	[35]
R_f_D (Cu)	mg/kg-day	0.04	[36,37]
R_f_D (Pb)	mg/kg-day	0.004	[35,38]
R_f_D (Ni)	mg/kg-day	0.02	[29]
C_sf_ (Cd)	(mg/kg-day)^−1^	0.38	
C_sf_ (Ni)	(mg/kg-day)^−1^	1.7	
C_sf_ (Pb)	(mg/kg-day)^−1^	0.0085	

### 2.8. Obtaining Cherry Pomace Powder

The “Van” cultivar was selected for pomace production based on its more favorable overall profile, showing higher phytochemical content and antioxidant activity, together with suitable physicochemical characteristics and acceptable heavy metal levels compared with “Stella”. Sweet cherries var. “Van” were sorted, washed with distilled water, and pre-crushed to remove the pits before pressing. Cherry pomace was obtained during juice extraction using a juicer (Philips HR1855, Royal Dutch Philips Electronics, Amsterdam, The Netherlands). The resulting pomace was packed in plastic bags and stored at −20 °C until freeze-drying. Drying was performed by lyophilization using a BIOBASE BK-FD10T freeze dryer (Biobase Biodustry Co., Ltd., Jinan, China) at −42 °C and 10 Pa for 48 h. The dried pomace had a final measured moisture content of 7.9% and was then ground using an MC 12 grinder (Stephan GmbH, Berlin, Germany). The powder was further milled for 50 s, yielding a mean particle size of 450–500 μm. Finally, the obtained powder was packed in a polymer film and stored under dry conditions at 20 °C until further characterization and use [39].

### 2.9. Characterization of Cherry Pomace Powder

#### 2.9.1. Proximate Composition of Cherry Pomace Powder

The proximate composition of the cherry powder, including moisture, ash, protein, fat, fiber, and carbohydrates (calculated by difference), was determined according to AOAC standard methods [40]. All analyses were performed in triplicate, and the results were expressed as g/100 g dry weight (DW). Protein content was determined by the Kjeldahl method using a conversion factor of N × 5.7, lipid content was measured by Soxhlet extraction, and ash content was determined by incinerating 5 g of sample in a muffle furnace at 600 °C for 4 h, followed by cooling in a desiccator and reweighing. Moisture content was measured by oven drying at 105 °C, while crude fiber was quantified using the enzymatic–gravimetric method described by Chantaro et al. [41]. Total carbohydrate content was calculated by difference using the following equation:

(6) [100 − (moisture + protein + fat + ash + fiber) %]

#### 2.9.2. Color Analysis of Cherry Pomace Powder

The color characteristics of the cherry pomace powder were measured using a Minolta Chroma Meter CR-410 (Konica Minolta, Osaka, Japan), previously calibrated with a standard white plate according to the manufacturer’s instructions. The recorded color parameters were L* (lightness), a* (red–green coordinate), and b* (yellow–blue coordinate). Additionally, the hue angle and chroma, which describe color tone and saturation intensity, respectively, were calculated [42].

#### 2.9.3. Mineral Composition

Zn, Cu, Fe, Mg, K, Na, Ca, and Mn content in cherry pomace powder was determined by atomic absorption spectroscope (ContrAA 700, Analytik Jena, Jena, Germany) according to Stoica et al. [39] method, and values were expressed in mg/100 g dw.

#### 2.9.4. Extraction of Biologically Active Compounds

Bioactive compounds from cherry pomace powder were extracted by ultrasound-assisted extraction according to Milea et al. [43], with minor modifications. One gram of powder was mixed with 10 mL of 70% ethanol containing 1% citric acid (1:9, acid/solvent), sonicated in an Elmasonic S 180 H bath (Elma, Singen, Germany) for 30 min at 40 kHz and 100 W at 40 ± 5 °C, and then centrifuged for 10 min at 6000 rpm and 4 °C. The supernatant was collected for phytochemical analyses.

#### 2.9.5. Total Anthocyanin Content

The total anthocyanin content was determined spectrophotometrically by the pH differential method [44]. For the analysis, the extract was diluted separately with buffer solutions at pH 1.0 (0.025 M potassium chloride) and pH 4.5 (0.4 M sodium acetate). Absorbance was measured at 520 and 700 nm using a UV–VIS spectrophotometer (Analytik Jena Specord 210 Plus, Jena, Germany). The results were expressed as milligrams of cyanidin-3-glucoside equivalents per gram of dry weight (mg C3G/g dw).

#### 2.9.6. Determination of Total Phenolic Content

For the determination of total phenolic content, 500 μL of extract was transferred into test tubes, followed by the addition of 2.5 mL Folin–Ciocalteu reagent and 2 mL of 7.5% sodium carbonate solution. The mixtures were allowed to react at room temperature for 60 min. Absorbance was then measured at 765 nm using a UV–VIS spectrophotometer (Analytik Jena Specord 210 Plus, Jena, Germany). The results were expressed as milligrams of gallic acid equivalents per gram of dry weight (mg GAE/g dw) [45].

#### 2.9.7. Determination of Total Flavonoid Content

Flavonoid content was determined using a modified aluminum chloride colorimetric method according to Pękal and Pyrzynska [46]. Briefly, 250 μL of extract (1 mg/mL) was mixed with 1.25 mL of deionized water and 0.075 mL of 5% NaNO_2_. After 5 min of incubation in the dark, 0.15 mL of 10% AlCl_3_ was added. Following an additional 6 min, 0.5 mL of 1 M NaOH and 0.775 mL of deionized water were added to the reaction mixture. Absorbance was measured at 510 nm using a UV–VIS spectrophotometer (Analytik Jena Specord 210 Plus, Jena, Germany). The flavonoid content was expressed as milligrams of catechin equivalents per gram of dry weight (mg CE/g dw).

#### 2.9.8. Determination of the Antioxidant Activity

The antioxidant activity of the extract was determined using the DPPH radical scavenging assay [47]. Briefly, 100 μL of extract was mixed with 3.9 mL of 0.1 mM DPPH methanolic solution and incubated in the dark at room temperature for 30 min. A blank was prepared by replacing the extract with methanol. Absorbance was measured at 515 nm using a UV–VIS spectrophotometer (Analytik Jena Specord 210 Plus, Jena, Germany). The results were expressed as percentage inhibition and μM Trolox equivalents per g dry weight (μM TE/g dw).

### 2.10. Dairy-Based Spreadable Product Preparation

To develop the enriched products and assess their functional potential, cherry pomace was incorporated at two levels, 5% (C1) and 10% (C2). These concentrations were selected based on previous studies and nutritional considerations, with the aim of improving both the functional value and sensory quality of the final products. The selected inclusion levels also allowed a systematic evaluation of their impact on the physicochemical and sensory properties of the dairy-based spreadable product formulations.

Three dairy-based spreadable product variants were prepared: a control sample without cherry pomace addition and two experimental samples containing 5% and 10% cherry pomace powder, respectively. The formulations, expressed as % (g/g), were as follows: spreadable cream cheese (72.25/69.60%), milk (10.50/9.60%), water (11/9%), cherry pomace powder (5/10%), salt (1.0/1.6%), and lemon juice (0.15/0.20%).

Sample preparation was carried out in several steps. First, the spreadable cream cheese and milk were vigorously homogenized using a Philips HR2100/40 blender (Philips, Amsterdam, The Netherlands) for 5 min at 1000 rpm. The cherry pomace powder was then added to the mixture and homogenized for 3 min at 1000 rpm. Next, the mixture was incorporated with salt, water, and lemon juice. The final mixture was left to stand for 5 min, then transferred into glass containers, covered with aluminum foil, and stored at 4 °C until analysis.

### 2.11. Characterization of Dairy-Based Spreadable Products

#### 2.11.1. Proximate and Phytochemical Composition

The value-added dairy-based spreadable samples were physicochemically characterized according to AOAC guidelines [48]. Their proximate composition, including moisture, crude protein, crude fiber, lipids, carbohydrates, and ash, was determined using the same standard procedures. In addition, the phytochemical profile of the dairy-based spreadable product enriched with cherry pomace powder was evaluated by determining total anthocyanin, total phenolic, and total flavonoid contents, and antioxidant activity using the corresponding analytical methods.

#### 2.11.2. Color Analysis

Dairy-based spreadable samples were characterized for CIELAB color parameters using a portable colorimeter with illuminant C (Chroma Meter CR-410, Konica Minolta, Osaka, Japan), previously calibrated with a white standard tile before each measurement. The measured parameters included L* (lightness), a* (red/green coordinate), and b* (yellow/blue coordinate). Chroma, hue angle, and total color difference (ΔE) were subsequently calculated according to Murariu et al. [49].

#### 2.11.3. Texture Analysis

The textural characteristics of the dairy-based spreadable samples containing cherry pomace powder were determined using a Brookfield CT3 texture analyzer (Brookfield Ametek, Middleboro, MA, USA). Cylindrical samples (10 mm diameter, 20 mm height) were prepared using a piston mold and subjected to texture profile analysis (TPA) with a 25.4 mm cylindrical probe. A double-compression cycle was applied without holding time to 50% deformation at a crosshead speed of 1 mm/s, using a trigger force of 0.067 N and a 9.8 N load cell. The parameters measured were firmness, adhesiveness, cohesiveness, springiness, and chewiness, as described by Frunză et al. [50]. Data were recorded and analyzed with TexturePro CT V1.5, and all measurements were carried out in triplicate.

#### 2.11.4. Scanning Electron Microscopy Analysis

Sample morphology and elemental composition were characterized by SEM–EDS using a Quanta 450 scanning electron microscope (FEI, Thermo Fisher Scientific, Hillsboro, OR, USA) equipped with an EDAX detector (AMETEK Inc., Berwyn, PA, USA). Samples were mounted on aluminum stubs as an approximately 2 mm-thick layer and analyzed after calibration with a standard Al/Cu reference sample. EDS spectra were processed with TEAM V4.1 software (EDAX Inc., Berwyn, PA, USA). Measurements were performed under low vacuum at approximately 6.1 × 10^−4^ Pa, using an accelerating voltage of 15 kV and 500× magnification [51].

#### 2.11.5. Sensorial Analysis

Sensory evaluation of the dairy-based spreadable samples was performed by 25 untrained panelists from the Department of Food Technologies, aged 18–39 years (40% male, 60% female). The sensory protocol was approved by the Ethics Committee of the Faculty of Agricultural Sciences, “Ion Ionescu de la Brad” Iasi, University of Life Sciences, Romania (approval no. 724/16 April 2026). Sensory evaluation was conducted in a dedicated sensory laboratory at “Ion Ionescu de la Brad” Iași University of Life Sciences, designed and operated in accordance with ISO 8589 [52] requirements for sensory test rooms. The attributes evaluated were appearance, odor, color, taste, aroma, aftertaste, hardness, adhesiveness, chewability, and overall acceptability, using a 7-point hedonic scale (1 = extremely dislike; 7 = extremely like) [39]. Sensory samples were served to the panelists on the day of their preparation. The panelists evaluated the samples at their own pace, following the coded serving order provided on the evaluation form. Bread and drinking water were available between samples for palate cleansing, and panelists were instructed to take a short pause between successive evaluations to reduce carry-over effects. Because the samples were presented in transparent containers, color perception was controlled by conducting the sensory evaluation in an ISO 8589-compliant sensory analysis laboratory under uniform lighting conditions. All samples were served in identical transparent plastic containers and assessed under the same environmental conditions to minimize external influences on color perception. The samples were labeled with random three-digit codes generated using a random-number procedure. The generated codes were checked before sample presentation to ensure that no duplicate or sequential codes were used.

### 2.12. Data Analysis

All data are presented as the mean ± standard deviation of triplicate determinations. The evaluated parameters were subjected to one-way analysis of variance (ANOVA) to determine differences among sample means. When significant effects were detected (*p* < 0.05), Tukey’s multiple range test was applied for post hoc comparison at the 5% significance level using SPSS statistical software (version 20.0, IBM Corp., Armonk, NY, USA). Principal component analysis (PCA) was performed on the sensory data using XLSTAT, an add-in software for Microsoft Office Excel (Trial Version 2024, Addinsoft, Paris, France).

## 3. Results and Discussion

### 3.1. Soil-Climate Conditions as Determinants of Fruit Quality

Climatic conditions recorded during the April–June interval (18.02 °C and 199.6 mm) fall within a range generally considered favorable for sweet cherry development, supporting both flowering progression and fruit growth. Such thermal conditions are known to enhance carbohydrate accumulation and fruit enlargement, while moderate precipitation ensures adequate water availability without inducing excessive physiological stress [53].

The soil physicochemical characteristics (Table 2) further support this favorable production context. The slightly acidic to neutral pH (6.79) is optimal for nutrient solubility and uptake, particularly for phosphorus and micronutrients, thus facilitating efficient root functioning [10,54]. The relatively high levels of total nitrogen (0.215%), available potassium (374 mg kg^−1^), and phosphorus (94 mg kg^−1^) indicate a well-supplied nutrient environment, which is directly associated with improved fruit size and soluble solids accumulation [55]. Similar relationships have been reported by Gonçalves et al. [56], who observed increased total soluble solids (SSC) and fruit weight under potassium-sufficient conditions.

The organic carbon (2.50%) and humus content (4.31%) suggest a structurally stable soil with good water retention capacity, which is essential under fluctuating climatic conditions. In addition, the elevated concentrations of Ca (2145 mg kg^−1^) and Mg (276 mg kg^−1^) indicate a balanced cationic status. This is relevant because calcium is associated with cell wall stabilization and maintenance of fruit firmness, while magnesium supports metabolic processes. In sweet cherry, both mineral nutrition and cultivar background are known to contribute substantially to final fruit quality [57].

### 3.2. Physical–Chemical Analysis of Cherry Fruits

For cherry trees, this physical characteristic varies even within the same variety, depending on the rootstock, the age of the trees, the agricultural practices employed, soil and climate conditions, fruit load, and other factors [58].

The two cultivars showed clear differences in their physicochemical profile, despite being grown under the same pedoclimatic conditions, indicating a strong genotype effect. Fruit weight ranged from 6.94 g in “Van” to 9.51 g in “Stella” (Table 3). These values fall within the interval reported for 23 Portuguese sweet cherry cultivars (4.9–11.8 g), which places both cultivars within the normal commercial range for sweet cherry fruit mass [59]. In the more recent Romanian study of several sweet cherry cultivars, “Van” reached 7.76 g fruit weight and 24.25 kg tree^−1^ yield, values very close to those observed in this study for “Van” (6.94 g and 25.12 kg tree^−1^), suggesting that the productivity pattern of this cultivar is consistent across temperate cultivation conditions [60]. The current trend is to breed cherry varieties with the largest possible fruit, which has also led to changes in the size classification categories. Thus, while fruits weighing over 6 g were considered large [61], current studies suggest that new varieties should have fruits weighing 11–13 g [10,62].

The level of soluble sugars (sucrose, glucose, and fructose) in fruit is an important quality parameter due to its significant influence on fruit flavor, and it also serves as an indicator of ripeness for consumption [63]. The total soluble solids content (SSC) was high in both cultivars (17.64–18.41 °Brix), confirming good sugar accumulation. These values are very close to those reported for “Van” in the recent Romanian dataset (17.85 °Brix), and they are fully consistent with the typical range described for high-quality sweet cherries grown under temperate conditions. Thus, although “Stella” had larger fruits, it also maintained slightly higher SSC, which suggests efficient assimilate accumulation rather than dilution by fruit size increase [60]. Similar scientific studies conducted in various climatic regions around the world have shown that, in the case of cherries, sugar content ranges from 11 to 25 °Brix during the ripening period [64,65,66], and only cherries with a soluble solids content greater than 18 °Brix are considered to be of superior quality [67].

Total acidity and pH are genetically determined qualitative characteristics, and their variability depends primarily on the variety and climatic factors during fruit development [68]. Changes in acidity are also reflected in the pH value of the vacuolar juice.

The pH values recorded in the present study (3.58 in “Stella” and 3.86 in “Van”) indicate moderate acidity and are close to the values recently reported specifically for these two cultivars during storage experiments, where pH ranged from 3.45 in “Van” to 3.66 in “Stella” at the beginning of storage. More broadly, sweet cherries are generally described as mildly acidic fruits, with pH values usually ranging between 3.7 and 4.2 [69].

Titratable acidity ranged from 0.69 to 0.78 mg malic acid 100^−1^, which is in very good agreement with values reported for sweet cherry cultivars from Iasi, Romania, where TA ranged between 0.5 and 0.9 g malic acid 100^−1^ g. The same Romanian source also showed that cultivar strongly influences the sugar-to-acid balance, which supports the present observation that “Van”, despite slightly lower SSC, exhibited higher acidity and therefore a more pronounced fresh taste profile. Similar studies conducted on different cherry varieties have shown that titratable acidity varied significantly between 0.39 and 0.87 mg malic acid 100 g^−1^ [70,71], and according to the study conducted by Crisosto et al. [72], the recommended range for titratable acidity in fully ripe cherries is 0.5 to 1.0 mg malic acid·100 g^−1^. Cherries are fruits valued by consumers both for their delicious taste and for their numerous nutritional properties with health benefits, such as their high vitamin C content [21,73].

Vitamin C (L-ascorbic acid) is the primary vitamin synthesized by plants and varies widely in fruits depending on species, variety, and soil and climate conditions [74]. Cherries are important sources of vitamin C, whose content ranges from 7.0 to 62.42 mg/100 g fresh fruit [75,76,77].

The evaluated cherry varieties had an average vitamin C content ranging from 7.72 mg/100 g in “Stella” to 9.03 mg/100 g in “Van”. In contrast to the reports by Średnicka-Tober et al. [78], which indicate that cherries can be characterized by a significantly higher vitamin C content, up to 42.89 mg·100 g^−1^, there are also studies [66,79] according to which, depending on the variety and the weather conditions of the study year, the average vitamin C content in cherries can range between 7.0 and 20.0 mg·100 g^−1^, values similar to those found in the current study. During the fruit growth and ripening period, a complex process of vitamin biosynthesis takes place, strongly influenced by eco-pedoclimatic conditions. Overall, the data indicate that “Stella” is characterized by larger and firmer fruits, whereas “Van” combines higher productivity with higher acidity and vitamin C, confirming that cultivar identity is a major determinant of fruit quality expression in sweet cherry.

Color is one of the most important indicators of fruit quality, of great interest to consumers as well as for determining marketing strategies (fresh consumption or processing) [80]. In cherries, color is a varietal characteristic and is determined by the presence of pigments in all cells of the epidermis and hypodermis [81].

Depending on the color changes that occur during the fruit ripening process, cherries can be white, white speckled with pink, yellow, red, bicolored (yellow and red), black, or brick-red [82]. The most commonly used varieties range in color from yellow to dark red. The characteristic color of ripe fruits is due to the biodegradation of chlorophyll pigments and the biosynthesis of anthocyanins (red fruits) and flavonoid pigments (yellow or bicolored fruits), and is primarily regulated at the transcriptional level [83]. Currently, varieties with light red, intense, glossy skin are preferred over dark red to black varieties, which have an overripe appearance [1].

The “Van” variety exhibited higher a* and C* values than “Stella”, indicating a more intense and saturated red color, consistent with its higher anthocyanin content. Studies on sweet cherry ripening have shown that a*, chroma, and hue angle are closely related to anthocyanin development and are reliable markers of color changes associated with ripening [9]. The relatively low L* values recorded for both varieties indicate dark-colored fruit, while the higher a* value for “Van” is consistent with this variety’s stronger red hue. In previous studies on sweet cherries, anthocyanin concentrations ranged from approximately 5 to 86 mg/100 g FW in one year and from 6 to 230 mg/100 g FW in another, and these changes were reflected in the CIELAB color parameters, particularly a* and the hue angle [84].

### 3.3. Phytochemical Properties of Cherry Extracts

Polyphenols are compounds widely found in horticultural products, whose importance stems from their physiological and biochemical properties, such as antioxidant activity, the ability to inhibit nitrosamine formation and regulate enzyme activity, the reduction of reactive oxygen species, and anticarcinogenic effects [85].

Multiple scientific studies on cherries have reported concentrations of phenolic compounds ranging from 58.31 to 115.41 mg gallic acid/100 g [86], 78.8 mg gallic acid equivalent/100 g fruit [87], 87.9 mg gallic acid equivalent/100 g fruit weight [10], 117.22–196.98 mg gallic acid equivalent/100 g fruit [88], and Kelebek & Selli (2011) [89] noted that they obtained a content ranging from 88.7 to 239.5 mg gallic acid equivalent/100 g in cherries.

The phytochemical results (Table 4) indicate a superior bioactive profile in “Van”, which showed higher antioxidant activity, total polyphenols, flavonoids, and anthocyanins than “Stella”. This pattern is not only internally consistent but also well supported by the literature showing marked cultivar-dependent variability in sweet cherry phenolic composition. In sweet cherries from Vojvodina, total polyphenols ranged from 4.12 to 8.34 mg GAE/g dw, total flavonoids from 0.42 to 1.56 mg/g dw, and total anthocyanins from 0.35 to 0.69 mg C3G/g dw. Against that background, the values obtained in this study for “Stella” and especially “Van” are comparatively higher, particularly for total polyphenols (9.33–10.47 mg GAE/g dw), flavonoids (1.69–2.75 mg CE/g dw), and anthocyanins (0.91–1.23 mg C3G/g dw), which places material at the upper end of reported sweet cherry phytochemical richness [90].

This variation in phenolic content was attributed to the fact that it can vary within a single fruit [91] or increase during ripening, likely due to an increase in anthocyanin content [65].

The antioxidant activity data reinforce this interpretation. In the recent Romanian cultivar study, “Van” showed a DPPH inhibition of 45.8%, whereas in the present study, antioxidant inhibition reached 91.11% in “Van” and 85.68% in “Stella”. Although direct comparison should be made cautiously because the extraction protocol, expression basis, and sample preparation may differ, the magnitude of the present values supports the conclusion that the investigated samples possess a strong antioxidant potential [60].

These data also agree with the broader evidence that phenolic content and antioxidant activity in sweet cherry are strongly cultivar-dependent and closely associated with the anthocyanin-rich fraction of darker fruits. In recent compositional studies, anthocyanins and hydroxycinnamic acids represented the dominant phenolic classes in sweet cherry fruit, and cultivars differed substantially in their total phenolic accumulation [92].

### 3.4. Concentrations of Heavy Metals in Orchard Cherry

**Figure 2:**
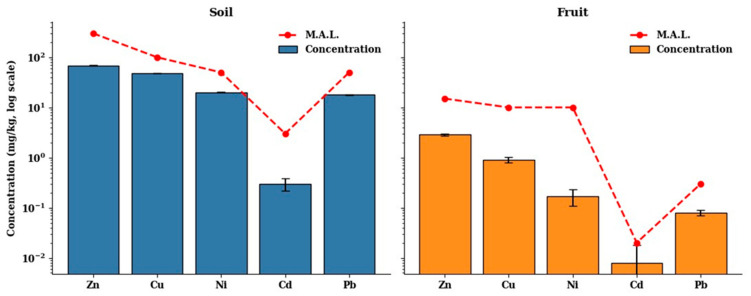
Heavy metals in soil and fruits relative to regulatory limits.

The soil concentrations of Zn, Cu, Ni, Cd, and Pb remained below the upper guideline values listed in Figure 2, indicating that the orchard environment cannot be considered heavily contaminated. This is important because soil contamination status is the first filter controlling elemental transfer to edible tissues. In contrast with contaminated horticultural environments, where elevated soil loads frequently translate into higher dietary exposure, the present orchard appears to provide a comparatively safe production background. Similar conclusions were reported for non-polluted reference areas in Romania, where fruits and vegetables showed substantially lower dietary risk indicators than those produced in mining-affected zones. Heavy metals, including Zn (69.11 mg kg^−1^) and Cu (47.87 mg kg^−1^), are involved in key enzymatic and redox processes and contribute to pigment synthesis and overall fruit quality. The availability of these elements is of particular relevance in the context of soil–plant interactions, as it governs their transfer and accumulation in fruit tissues, with direct implications for both nutritional value and safety-related considerations [93]. Cherries are rich in minerals, with average levels ranging from 0.19% to 0.62%. The main minerals found in cherries are: potassium, calcium, magnesium, iron, copper, manganese, and zinc [1].

At the fruit level, all measured concentrations were below the maximum allowable limits (Figure 2). This is particularly relevant for Cd and Pb, whose concentrations in the present fruits were 0.008 and 0.08 mg/kg, respectively. These values are clearly lower than those reported in an earlier Romanian study on *Prunus* fruits from an urban environment, where cherry Cd ranged from 0.123 to 0.431 mg/kg dry weight, and Pb was not detected. Even though the basis of expression differs between studies, the comparison still suggests that the present material is characterized by low contaminant burden, especially for cadmium. For Cu, your fruit value (0.91 mg/kg) is also well below the 3.09–4.13 mg/kg dry weight reported for urban-grown cherries in that study [94].

At the same time, Zn and Cu should not be interpreted exclusively through a toxicological lens. Both are essential micronutrients involved in enzymatic activity and redox metabolism, and their presence at moderate concentrations may be compatible with normal plant physiological functioning. What matters in the food-safety context is not only their presence, but whether accumulation in fruit exceeds admissible thresholds, which is not the case here [95].

### 3.5. Health-Risk Analysis of Heavy Metals Through Cherry Consumption

The contamination risk (ContR) values for Zn, Cu, Ni, Cd, and Pb in cherry fruits (Table 5) were all below 1.0, indicating no significant contamination and confirming their suitability for safe consumption.

The health-risk assessment confirms the food-safety relevance of the analytical results. All THQ values were far below 1, and the cumulative HI was 3.18 × 10^−2^, also well below the critical threshold of 1. These values indicate the absence of significant non-carcinogenic risk associated with cherry consumption under the exposure assumptions used in the study. This interpretation is fully consistent with risk-assessment frameworks applied in the literature, including the Romanian case study from the Banat area, where lower-risk horticultural products from the reference area also generated THQ/TTHQ values below the concern threshold [96].

The carcinogenic risk values calculated for Ni, Cd, and Pb were also extremely low, all below 10^−6^–10^−7^ order of magnitude, indicating negligible carcinogenic concern. From an ISI discussion perspective, this is an important strength of the manuscript: the analytical determination of heavy metals is not left at the descriptive level, but is integrated with an exposure-based interpretation, which substantially increases the relevance of the fruit safety assessment [96].

### 3.6. Global Characterization of Cherry Pomace Powder

Table 6 presents the phytochemical composition, color, mineral, and nutritional characteristics of the extract derived from cherry pomace powder.

Cherry pomace powder exhibited a composition typical of a bioactive fruit-processing by-product, characterized by high total polyphenols (1046.80 mg GAE/100 g), appreciable flavonoid (275.26 mg CE/100 g), and anthocyanin (123.27 mg C3G/100 g) contents. The antioxidant activity (21.43 ± 1.25 µM TE/g) further supports the presence of a bioactive phytochemical matrix. Color measurements (L* = 50.53, a* = 8.93, b* = 7.02) placed the powder in the red-yellow region, consistent with the retention of fruit pigments after processing. When compared with optimized sour-cherry-pomace extraction reported by Yılmaz et al. [97], the present total polyphenol content is lower than the reported 14.23 ± 0.38 mg/g (≈1423 mg/100 g), but the anthocyanin level is higher than their 0.41 ± 0.02 mg/g (≈41 mg/100 g). In addition, cultivar-specific work by Ciccoritti et al. [98] showed that total monomeric anthocyanins in sour cherry pomace may vary widely, from 0.22 ± 0.1 mg/g dw to 4.3 ± 0.1 mg/g dw, confirming that the present anthocyanin content lies within the broad variability expected for cherry pomace matrices.

Proximate analysis showed that the material was predominantly composed of total dietary fiber (49.83 g/100 g) and carbohydrates (27.56 g/100 g), while protein (8.42 g/100 g) and fat (3.37 g/100 g) were comparatively low, confirming the potential of this powder as a fiber-rich functional ingredient. The moisture content (8.11 g/100 g) suggests good storage stability, whereas the ash value (2.71 g/100 g) indicates the presence of an appreciable mineral fraction. Gumul et al. [99] reported sour cherry pomace with 13.57% protein, 3.02% fat, 10.97% carbohydrates, and 48.7% dietary fiber (4.5% soluble and 44.2% insoluble), while a later study on dried, pitted, fractionated sour cherry pomace reported 5.76–6.71% moisture and 1.06–3.02% ash depending on particle-size fraction. Jūrienė et al. [100].

Potassium was the predominant mineral (329.28 mg/100 g), followed by calcium and magnesium, while sodium and iron remained very low. Pomace mineral composition differs markedly across species, largely because each species has a unique biochemical profile that influences the concentration of potassium, calcium, phosphorus, and other essential micronutrients. Reported variations in the literature have also been associated with factors such as cultivar, climatic conditions, soil properties, processing parameters, and extraction techniques [101].

The results characterize cherry pomace powder as a low-moisture ingredient with a high dietary fiber and phenolic content, a mineral profile dominated by potassium, moderate protein levels, and low fat content, together with a stable reddish chromatic profile. Collectively, these properties highlight its potential as a value-added functional food ingredient, particularly in formulations aimed at improving dietary fiber content, bioactive compound availability, mineral enrichment, and natural color attributes.

### 3.7. Physicochemical, Phytochemical, and Antioxidant Activity Characterization of Value-Added Dairy-Based Spreadable Samples

**Figure 3:**
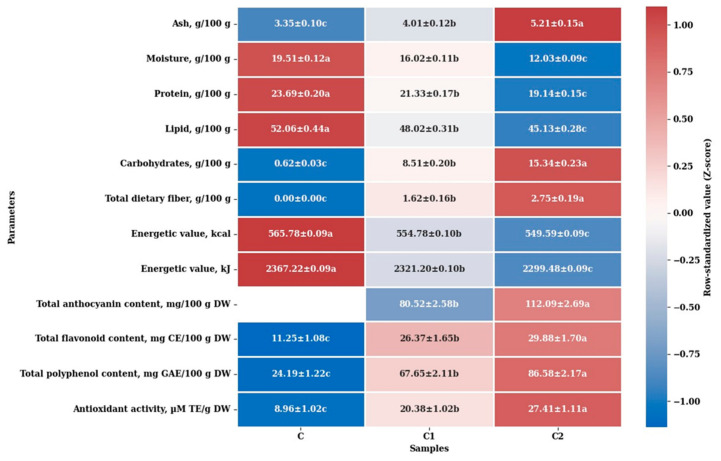
Physicochemical, phytochemical, and antioxidant profile of dairy-based spreadable samples (mean values followed by at least one identical letter are not significantly different (*p* > 0.05)).

The incorporation of cherry pomace powder significantly affected the physicochemical composition of dairy-based spreadable samples (Figure 3). The control sample (C) differed significantly from dairy-based spreadable enriched with 5% (C1) and 10% (C2) cherry pomace powder (*p* < 0.05). The results demonstrate a clear concentration-dependent effect of pomace addition, with progressive compositional changes as the supplementation level increased from 5 to 10%.

Compared with the control sample (C), the addition of 5% (C1) and 10% (C2) pomace powder significantly increased ash, carbohydrates, and total dietary fiber, while significantly decreasing moisture, protein, lipid, and energetic value. Ash increased from 3.35 to 5.21 g/100 g, indicating mineral enrichment, whereas moisture decreased from 19.51 to 12.03 g/100 g, reflecting a progressive increase in total solids. The addition of cherry pomace powder also resulted in a significant decrease in protein content, from 23.69 ± 0.20 g/100 g in C to 21.33 ± 0.17 g/100 g in C1 and 19.14 ± 0.15 g/100 g in C2. This represents reductions of approximately 10.0% and 19.2%, respectively. A similar trend was observed for lipid content, which decreased from 52.06 ± 0.44 g/100 g in the control to 48.02 ± 0.31 g/100 g and 45.13 ± 0.28 g/100 g in C1 and C2, respectively. In contrast, carbohydrates rose from 0.62 to 15.34 g/100 g, and total dietary fiber increased from 0.00 to 2.75 g/100 g, confirming the contribution of cherry pomace powder as a source of plant-derived solids and dietary fiber. The energetic value decreased from 565.78 to 549.59 kcal/100 g, mainly due to the reduction in lipid content.

The progressive increase in ash, carbohydrates, and total dietary fiber in the samples obtained in the study is mechanistically compatible with the addition of pomace, as fruit pomace contributes minerals, structural polysaccharides, and non-milk solids to the dairy-based matrix [102,103]. In the Frühbauerová et al. [104] study, they produced processed cheese spread containing 1% and 2% grape-skin powder derived from grape pomace. In that study, protein content increased significantly at the 2% addition level, from 112.5 g/kg in the control to 128.4–129.7 g/kg, while dry matter also tended to increase, reaching 419.0 g/kg in the sample with 2% oven-dried powder; by contrast, fat and ash did not change significantly.

Cherry powder addition significantly enhanced the phytochemical profile and antioxidant activity of dairy-based spreadable samples (*p* < 0.05) (Figure 3). The data further reveal a clear dose-dependent effect, with the highest values consistently observed in the sample containing 10% (*w*/*w*) cherry powder (C2).

As expected, total anthocyanin content was not detected in the control sample, whereas the cherry-enriched samples showed substantial anthocyanin levels, reaching 80.52 ± 2.58 mg/100 g DW in C1 and 112.09 ± 2.69 mg/100 g dw in C2. The increase from C1 to C2 was approximately 39.2%, confirming that anthocyanins were directly introduced through cherry-powder addition and accumulated proportionally with increasing fortification level. Total flavonoid content increased from 11.25 in the control to 26.37 and 29.88 mg CE/100 g DW in C1 and C2, respectively, while total polyphenols rose from 24.19 to 67.65 and 86.58 mg GAE/100 g dw. The pronounced rise in total polyphenols confirms that cherry powder acted as a major source of phenolic compounds in the fortified dairy-based spreadable samples. The same dose-dependent trend was observed for antioxidant activity, which rose from 8.96 ± 1.02 µM TE/g dw in the control to 20.38 ± 1.02 µM TE/g dw in C1 and 27.41 ± 1.11 µM TE/g dw in C2. This corresponds to increases of approximately 127.5% and 205.9%, respectively, relative to the control.

In the study by Lucera et al. [102], the researchers fortified a dairy-based spreadable with 5% red grape pomace. In that work, pomace addition markedly improved the functional profile of the cheese: total phenolic content increased from 0.66 mg GAE/g dw in the control to 2.34 mg GAE/g dw in red-grape-pomace cheese. Antioxidant activity also increased substantially, with ABTS values rising from 0.96 in the control to 3.95–4.00 mg TE/g dw. Frühbauerová et al. [104] reported that the phenolic profile and antioxidant capacity of processed cheese spread improved after enrichment and that freeze-dried grape-skin powder performed better than oven-dried powder for total phenolics and ABTS radical scavenging. The total phenolics increased from 0.13 ± 0.02 in the control to 0.47 ± 0.07 and 0.54 ± 0.07 mg GAE/g dry matter in cheeses enriched with oven-dried and freeze-dried grape-skin powder, respectively. Therefore, the antioxidant effect of the enriched cheese depended on both the antioxidant load of the by-product and the treatment applied to that by-product before incorporation. The concomitant increases in flavonoids, total polyphenols, and antioxidant activity also agree with the broader dairy-based spreadable products literature, which reports that polyphenol-rich plant by-products consistently enhance the antioxidant profile of dairy-based spreadable formulations, although the final values vary with cultivar, geographic origin, treatment, and matrix interactions with dairy proteins. The results demonstrate that the enrichment of dairy-based spreadable with cherry powder significantly improves their mineral and fiber content and slightly reduces caloric density, phytochemical composition, and antioxidant activity in a concentration-dependent manner.

### 3.8. Colorimetric Parameters of Value-Added Dairy-Based Spreadable Samples

The incorporation of cherry powder significantly influenced the color attributes of dairy-based spreadable samples (Table 7). The data show that cherry powder induced a marked and concentration-dependent modification of the visual appearance of the dairy-based spreadable product, shifting the product from a light yellow-greenish dairy color toward a darker and more reddish hue.

Lightness (L*) decreased markedly from 90.55 in the control to 58.98 and 50.02 in C1 and C2, respectively, indicating progressive darkening of the dairy-based spreadable matrix. At the same time, a* values shifted from negative (−2.80) in the control to strongly positive values (13.57 and 16.77), demonstrating a pronounced increase in redness following cherry powder incorporation. In contrast, b* decreased from 14.56 to 4.37 and 3.99, showing a reduction in yellowness. Chroma remained statistically similar between C and C1, but increased significantly in C2, indicating greater color saturation at the higher supplementation level. The color modifications observed in the present study are consistent with the literature on fruit by-product enrichment in dairy-based formulation. A very similar trend was reported by Rațu et al. [105] for cheese supplemented with grape-skin powder. In that study, L* decreased from 68.31 ± 0.04 in the control to 53.33 ± 0.09 in the enriched cheese, a* increased from −1.22 ± 0.27 to 8.31 ± 0.06, and b* decreased from 11.59 ± 0.05 to 6.46 ± 0.08, with all differences significant at *p* < 0.05. Chroma also decreased slightly from 11.66 ± 0.05 to 10.53 ± 0.10. Thus, the direction of change in our samples is fully consistent with this grape-skin-cheese model, although the shift toward redness in C1 and especially C2 samples was even stronger. Comparable effects were also described in berry-by-product-enriched curd cheese, particularly with elderberry and blueberry by-products, which produced lower lightness and higher redness than the control. For example, elderberry by-product cheese showed L* = 70.2 ± 3.82, a* = 7.35 ± 0.88, and b* = 7.24 ± 0.49, while blueberry by-product cheese showed L* = 80.4 ± 3.21, a* = 1.69 ± 0.39, and b* = 8.26 ± 0.51 [106]. Overall, these findings confirm that cherry powder acts as a strong natural coloring agent in dairy-based spreadable and that the effect is concentration-dependent.

The large ΔE values (36.99 and 46.23) confirm that the color changes were highly perceptible and intensified with increasing cherry-powder concentration. Overall, cherry powder acted as an effective natural coloring ingredient, producing darker, redder, and more visually distinctive dairy-based spreadable samples.

From a technological and product-development perspective, these findings demonstrate that cherry powder functions not only as a source of bioactive compounds but also as a strong natural colorant in dairy-based spreadable formulations. The addition of 5% cherry powder was sufficient to cause substantial darkening and reddening, whereas 10% addition produced an even more intense and saturated red-toned product. Therefore, the color data support the conclusion that cherry powder has high potential for the development of visually differentiated value-added dairy spreads.

### 3.9. Texture Analysis of Value-Added Dairy-Based Spreadable Samples

**Figure 4:**
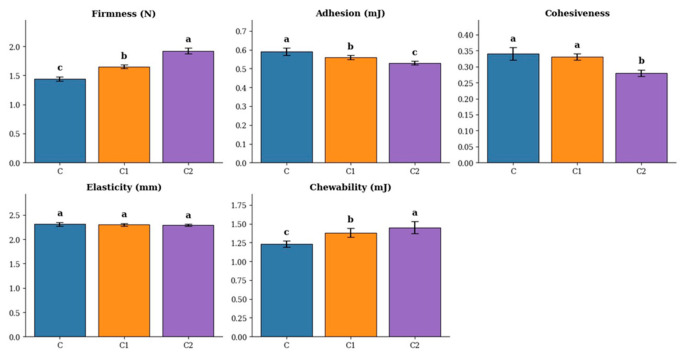
Textural parameters of dairy-based spreadable samples (mean values followed by different lowercase letters differ significantly (*p* < 0.05)).

The incorporation of cherry powder significantly influenced most of the textural properties of dairy-based spreadable samples (Figure 4). The results indicate that the addition of cherry powder progressively modified the dairy-based spreadable matrix, yielding samples that were firmer, less adhesive, and less cohesive, while maintaining similar elastic behavior.

Firmness increased progressively from 1.44 N in the control to 1.65 N in C1 and 1.92 N in C2, indicating that powder addition promoted the formation of a denser and more resistant matrix. In contrast, adhesion decreased significantly from 0.59 to 0.53 mJ, suggesting reduced stickiness with increasing enrichment. Cohesiveness remained unchanged at the 5% level but decreased significantly at 10%, indicating that higher powder incorporation may weaken matrix continuity. Elasticity was not significantly affected, showing that shape recovery properties were preserved across formulations. Chewability increased significantly in enriched samples compared with the control. The increase in chewability is consistent with the observed rise in firmness and the reduction in moisture, both of which contribute to a denser product requiring greater mechanical energy during oral processing. The textural behavior observed in the present study is in agreement with the literature showing that plant by-products can markedly modify dairy-based spreadable structure, although the direction of change depends on formulation. Previous work on fruit by-products shows that responses are matrix-dependent: wine-pomace cheeses have been reported to become softer and less spreadable, whereas other plant powders or fibers have either increased hardness or weakened cohesiveness by disrupting the cheese network. In particular, studies on date-seed-fortified processed cheese and blueberry-enriched spreadable processed cheese indicate that higher levels of fibrous plant material can impair internal matrix continuity, which agrees with the significant drop in cohesiveness observed here at the 10% fortification level. Thus, cherry powder appears to act as a structuring ingredient at lower addition levels, while at higher concentrations it begins to interfere with the continuity of the dairy-based spreadable matrix [107,108,109,110]. Overall, cherry-powder addition resulted in a firmer, less adhesive, and moderately less cohesive dairy-based spreadable matrix, while maintaining elastic behavior.

### 3.10. Scanning Electron Microscopy of Value-Added Dairy-Based Spreadable Samples

**Figure 5:**
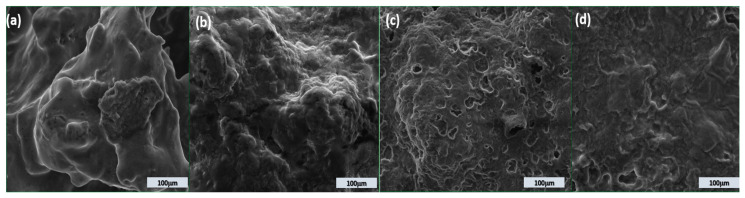
Scanning electron microscopy micrographs of cherry pomace powder (**a**); control sample without cherry pomace powder addition (C) (**b**); and dairy-based spreadable samples enriched with cherry pomace powder, containing 5% powder (C1) (**c**); and 10% powder (C2) (**d**), respectively.

The SEM micrographs presented in Figure 5 highlighted distinct morphological differences between the cherry pomace powder and the dairy-based spreadable samples. Figure 5a, corresponding to cherry pomace powder, showed an irregular, compact, and heterogeneous particle morphology, with rough surfaces and agglomerated structures, characteristic of a fibrous plant-derived material. Such structural features suggest the presence of insoluble cell wall components and fragmented pomace particles.

The control sample without powder addition (Figure 5b) exhibited a more continuous, compact, and homogeneous matrix, with a relatively smoother surface and fewer visible discontinuities. This microstructure indicates a well-organized dairy network, likely reflecting a more uniform distribution of the protein–fat phase in the absence of plant material.

In sample C1, Figure 5c, containing 5% cherry pomace powder, the matrix became more irregular and less compact than the control, with the appearance of pores, voids, and structural heterogeneity. These changes suggest that the incorporation of pomace particles disturbed the continuity of the native dairy matrix and promoted the formation of a more open network.

For sample C2, Figure 5d, containing 10% cherry pomace powder, the structural disruption appeared more pronounced, with a denser accumulation of irregular features and a more heterogeneous surface organization. The higher powder level seemed to intensify matrix discontinuity and reduce structural uniformity, indicating stronger interference of pomace particles within the dairy system.

Overall, the SEM observations suggest that increasing the level of cherry pomace powder progressively modified the microstructure of the spreadable product, shifting it from a compact and cohesive matrix toward a more heterogeneous, fibrous, and disrupted network. These structural modifications may be associated with enhanced functional properties, particularly in terms of texture, water-holding capacity, and sensory characteristics. The microstructural appearance of the fortified samples was comparable to that commonly described for processed cheese [111].

### 3.11. Sensory Analysis of Value-Added Dairy-Based Spreadable Samples

**Figure 6:**
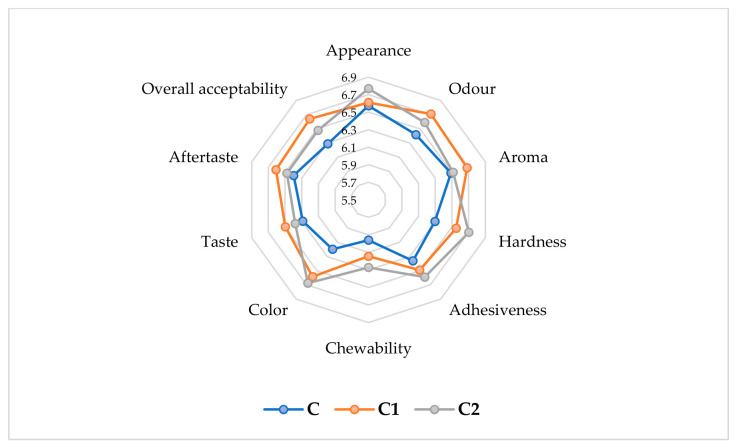
Comparative diagram of sensory attributes specific to the types of dairy-based spreadable: C—dairy-based spreadable without adding cherry powder, C1, and C2—dairy-based spreadable with 5 and 10% cherry powder.

From a sensory point of view, the dairy-based spreadable samples were analyzed using the following 10 attributes: appearance, odor, color, taste, aroma, aftertaste, hardness, adhesiveness, chewability, and overall acceptability using the scoring scale method from 1 to 7 (Figure 6).

Sensory evaluation showed that all samples received relatively close and favorable scores, ranging from 5.96 to 6.77. The enriched dairy-based spreadable samples generally performed better than the control, indicating a positive sensory effect of cherry-powder incorporation. C1 showed higher scores for odor, aroma, taste, aftertaste, and overall acceptability, suggesting that the 5% addition level provided the most balanced sensory profile. In contrast, C2 received the highest scores for appearance, hardness, adhesiveness, chewability, and color, indicating that the 10% addition level enhanced visual and textural perception more strongly.

The sensory results indicate that cherry-powder addition enhanced the acceptability of the dairy-based spreadable sample, with the 5% formulation (C1) appearing to be the most successful from an overall sensory standpoint. These results suggest that moderate fortification offered the best compromise between improved appearance and texture and desirable flavor perception.

**Figure 7:**
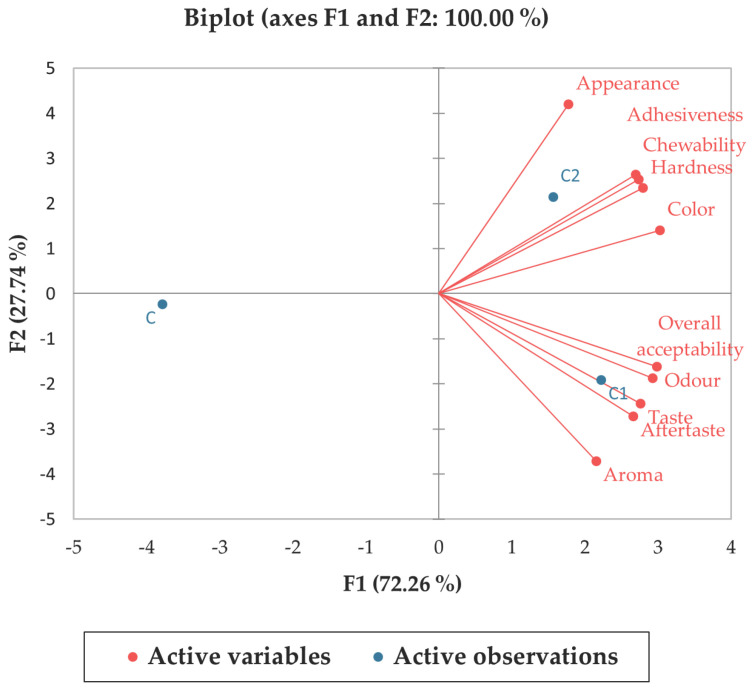
Illustration of Principal Component Analysis (PCA) biplot.

The PCA biplot (Figure 7) revealed a clear separation between the control sample and the cherry powder-enriched samples. The first two principal components explained 100.00% of the total variance, with F1 and F2 accounting for 72.26% and 27.74%, respectively. The control sample was located on the negative side of F1, whereas C1 and C2 were grouped on the positive side, indicating that cherry-powder addition was the main factor in this study influencing sensory differentiation. Sample C1 was more closely associated with aroma, taste, aftertaste, and overall acceptability, suggesting a more balanced and preferred sensory profile. In contrast, sample C2 was associated mainly with appearance, color, hardness, adhesiveness, and chewiness, indicating that the higher level of cherry powder exerted a stronger effect on visual and textural attributes. These results suggest that moderate cherry-powder incorporation improved consumer-related sensory characteristics, while the higher level enhanced structural and visual properties.

The sensory pattern observed in the present study is consistent with the scientific literature on fruit-enriched spreadable and processed cheeses. In a Petit Suisse-type dairy snack, the formulation containing 10 g/100 g blueberry syrup plus 2 g/100 g blueberry bagasse powder achieved the highest sensory scores, showing that fruit-derived ingredients can improve acceptance when used at an optimized level rather than simply at the maximum possible dose [105]. Similarly, in the present work, the 5% cherry-powder formulation showed the highest odor, aroma, taste, aftertaste, and overall acceptability scores, whereas the 10% formulation achieved the highest appearance, color, and texture-related scores. These findings suggest that moderate cherry-powder incorporation provides the most favorable balance between visual enhancement, textural modification, and flavor acceptance.

## 4. Limitations and Future Perspectives

Although this study provides an integrated assessment of orchard fruit quality, cherry pomace characterization, and its valorization in dairy-based spreadable products, several limitations should be acknowledged. The experiment was conducted under the pedoclimatic conditions of a single orchard and during one harvest season; therefore, the relationships observed among soil conditions, cultivar response, fruit quality, and by-product functionality should be validated across different years, locations, orchard management systems, and cultivars. In addition, cherry pomace powder was obtained only from the Van cultivar, which may limit the generalization of the results to other cherry genotypes with different phytochemical and technological profiles.

The dairy-based spreadable formulations were evaluated at two incorporation levels, 5% and 10%, allowing clear concentration-dependent effects to be identified. However, a broader range of inclusion levels should be investigated to optimize the balance between functional enrichment, texture, color, flavor, and consumer acceptability. Further research is also needed to assess shelf-life stability, microbiological safety, pigment and phenolic retention, and possible changes in antioxidant activity during refrigerated storage.

Another limitation is that sensory analysis was performed using untrained panelists. Although this approach provides useful information on consumer-oriented acceptability, future studies could combine consumer testing with trained descriptive sensory analysis to obtain a more detailed understanding of flavor, texture, and aftertaste changes induced by cherry pomace addition. Moreover, in vitro digestion and bioaccessibility studies would help clarify the release and potential availability of phenolic compounds, anthocyanins, minerals, and dietary fiber after incorporation into the dairy matrix.

From an application perspective, the results support the potential of dried cherry pomace as a functional ingredient for circular food innovation. Further work should focus on pilot-scale production, process optimization, economic feasibility, and environmental assessment to evaluate the practical implementation of this strategy within sustainable fruit-processing and dairy product value chains.

## 5. Conclusions

This study provides a comprehensive framework linking soil–orchard system characteristics with fruit quality expression and the functional valorization of cherry-derived by-products within a circular food innovation context. The results demonstrate that pedoclimatic conditions, particularly soil fertility status and balanced mineral composition, play a decisive role in shaping fruit physicochemical and phytochemical attributes.

Under the studied conditions, both cultivars exhibited distinct quality profiles, confirming the strong influence of genotype on fruit development, while maintaining values within the optimal range reported for high-quality sweet cherries. The observed levels of soluble solids, organic acids, and vitamin C indicate favorable metabolic performance, whereas the elevated phenolic and antioxidant contents, particularly in “Van”, highlight the functional potential of the fruit matrix.

The low concentrations of heavy metals in both soil and fruit, together with THQ and HI values below critical thresholds, confirm the safety of the production system and indicate limited soil-to-fruit transfer of potentially toxic elements. These findings reinforce the suitability of the studied orchard environment for producing safe and nutritionally valuable fruits.

Importantly, the study demonstrates that cherry pomace retains a significant proportion of bioactive compounds, including phenolics, anthocyanins, and dietary fiber, confirming its potential as a high-value functional ingredient. The incorporation of pomace powder into dairy-based spreadable formulations resulted in improved phytochemical composition, antioxidant activity, and mineral content, while also influencing color and textural properties in a concentration-dependent manner.

Taken together, these results support the concept that fruit quality and the functional potential of derived products are intrinsically linked to soil–orchard system characteristics. The integration of primary production factors with by-product valorization highlights a viable pathway for extending the cherry value chain and advancing circular bioeconomy strategies in sustainable horticultural systems.

## Figures and Tables

**Table 2:** Soil physicochemical properties (0–20 cm).

Properties	Unit Measure	Results
pH		6.79 ± 0.02
Total nitrogen (Nt)	%	0.215 ± 0.16
Phosphorus (P_2_O_5_)	mg kg^−1^	94 ± 0.32
Potassium (K_2_O)	mg kg^−1^	374 ± 0.21
Organic carbon (Corg)	%	2.50 ± 0.20
Humus	%	4.31 ± 0.22
Calcium (Ca)	mg kg^−1^	2145 ± 0.41
Magnesium (Mg)	mg kg^−1^	276 ± 0.25

**Table 3:** Physical–chemical characteristics of cherry fruits.

Parameters		Varieties	
		Stella	Van
Weight (g)		9.51 ± 0.21 ^a^	6.94 ± 0.19 ^b^
Stone (g)		0.49 ± 0.15 ^a^	0.42 ± 0.13 ^a^
Total soluble solids (°Brix)		17.64 ± 0.24 ^b^	18.41 ± 0.26 ^a^
Dry matter (%)		25.11 ± 0.25 ^a^	24.85 ± 0.28 ^b^
Pulp firmness (units HPE) (N/0.10 cm^2^)		51 ± 0.11 ^a^	43 ± 0.10 ^b^
pH		3.58 ± 0.08 ^a^	3.86 ± 0.07 ^a^
Titratable acidity (mg malic acid 100^−1^)		0.69 ± 0.09 ^a^	0.78 ± 0.10 ^a^
C vitamin (mg/100 g)		7.72 ± 0.22 ^b^	9.03 ± 0.25 ^a^
Yield/tree (kg)		19.21 ± 0.17 ^b^	25.12 ± 0.24 ^a^
Colorimetric parameters	L*	30.53 ± 0.23 ^a^	29.82 ± 0.22 ^b^
	a*	18.97 ± 0.18 ^b^	23.00 ± 0.20 ^a^
	b*	7.03 ± 0.16 ^b^	11.51 ± 0.17 ^a^
	C*	20.23 ± 0.14 ^b^	25.72 ± 0.18 ^a^
	h°	0.35 ± 0.01 ^a^	0.46 ± 0.01 ^a^

Means within the same row sharing at least one common superscript letter do not differ significantly at *p* > 0.05.

**Table 4:** Phytochemical properties of cherry fruits.

Phytochemical Properties		Varieties	
		Stella	Van
Antioxidant activity	µmol Trolox/g dw	18.52 ± 0.11 ^b^	19.79 ± 0.13 ^a^
	Inhibition %	85.68 ± 0.09 ^b^	91.11 ± 0.12 ^a^
Total polyphenols (mg GAE/g dw)		9.33 ± 0.13 ^b^	10.47 ± 0.15 ^a^
Total flavonoids (mg CE/g dw)		1.69 ± 0.08 ^b^	2.75 ± 0.10 ^a^
Total anthocyanins (mg C_3_G/g dw)		0.91 ± 0.05 ^b^	1.23 ± 0.08 ^a^

Means within the same row sharing at least one common superscript letter do not differ significantly at *p* > 0.05.

**Table 5:** Health-risk assessment of heavy metals through cherry consumption.

Heavy Metals	ContR	EDI	THQ	$\mathrm{HI}=\Sigma {\mathrm{THQ}}_{i}$	CR
Zn	41.24 × 10^−2^	1.35 × 10^−3^	3.65 × 10^−3^	3.18 × 10^−2^	-
Cu	19.01 × 10^−2^	4.32 × 10^−4^	1.08 × 10^−2^		-
Ni	8.44 × 10^−3^	8.07 × 10^−5^	4.04 × 10^−3^		1.37 × 10^−7^
Cd	2.67 × 10^−2^	3.80 × 10^−6^	3.80 × 10^−3^		1.44 × 10^−9^
Pb	4.47 × 10^−3^	3.79 × 10^−5^	9.49 × 10^−3^		3.28 × 10^−10^

**Table 6:** Comprehensive analysis of the cherry pomace powder.

Characteristics	Cherry Pomace Powder
Total anthocyanins, mg C3G/100 g dw	123.27 ± 0.34
Total flavonoids, mg CE/100 g dw	275.26 ± 0.51
Total polyphenols, mg GAE/100 g dw	1046.80 ± 1.64
Antioxidant activity, µM TE/g dw	21.43 ± 1.25
Ash, g/100 g dw	2.71 ± 0.28
Moisture, g/100 g dw	8.11 ± 0.17
Crude protein, g/100 g dw	8.42 ± 0.21
Crude fat, g/100 g dw	3.37 ± 0.14
Carbohydrates, g/100 g dw	27.56 ± 0.26
Total dietary fiber, g/100 g dw	49.83 ± 0.27
L*	50.53 ± 0.22
a*	8.93 ± 0.13
b*	7.02 ± 0.22
Hue angle	0.67 ± 0.02
Chroma	11.36 ± 0.11
Magnesium, mg/100 g	21.15 ± 0.19
Calcium, mg/100 g	40.06 ± 0.23
Potassium, mg/100 g	329.28 ± 0.69
Sodium, mg/100 g	1.86 ± 0.20
Copper, mg/100 g	0.49 ± 0.11
Zinc, mg/100 g	0.16 ± 0.09
Iron, mg/100 g	1.02 ± 0.14
Manganese, mg/100 g	0.26 ± 0.13

**Table 7:** Colorimetric parameters of dairy-based spreadable samples.

Samples of Dairy-Based Spreadable	L*	a*	b*	Chroma	Hue Angle	ΔE
C	90.55 ± 0.11 ^a^	−2.80 ± 0.06 ^c^	14.56 ± 0.10 ^a^	14.83 ± 0.12 ^b^	178.62 ± 0.02 ^a^	-
C1	58.98 ± 0.07 ^b^	13.57 ± 0.09 ^b^	4.37 ± 0.07 ^b^	14.26 ± 0.10 ^b^	0.32 ± 0.02 ^b^	36.99 ± 0.15 ^b^
C2	50.02 ± 0.04 ^c^	16.77 ± 0.08 ^a^	3.99 ± 0.06 ^c^	17.24 ± 0.14 ^a^	0.23 ± 0.02 ^b^	46.23 ± 0.17 ^a^

Mean values followed by at least one common letter in the same column are not significantly different (*p* > 0.05).

## Data Availability

The original contributions presented in the study are included in the article; further inquiries can be directed to the corresponding author.
